# Effect of periprocedural furosemide-induced diuresis with matched isotonic intravenous hydration in patients with chronic kidney disease undergoing transcatheter aortic valve implantation

**DOI:** 10.1007/s00392-023-02234-z

**Published:** 2023-06-01

**Authors:** Lisa Voigtländer-Buschmann, Sarina Schäfer, Christian Schmidt-Lauber, Jessica Weimann, Mina Shenas, Julian Giraldo Cortes, Piotr Mariusz Kuta, Tanja Zeller, Raphael Twerenbold, Moritz Seiffert, Niklas Schofer, Yvonne Schneeberger, Andreas Schäfer, Johannes Schirmer, Hermann Reichenspurner, Stefan Blankenberg, Lenard Conradi, Ulrich Schäfer

**Affiliations:** 1 Department of Cardiology, University Heart and Vascular Center Hamburg, Hamburg, Germany; 2 III. Department of Medicine, University Medical Center Hamburg-Eppendorf, Hamburg, Germany; 3 Institute for Clinical Chemistry and Laboratory Medicine, University Medical Center Hamburg-Eppendorf, Hamburg, Germany; 4 Department of Cardiovascular Surgery, University Heart and Vascular Center Hamburg, Hamburg, Germany; 5 Department of Cardiology, Heart and Vessel Center Bad Bevensen, Bad Bevensen, Germany

**Keywords:** Transcatheter aortic valve implantation, Acute kidney injury, RenalGuard, Chronic kidney disease

## Abstract

**Background:**

Acute kidney injury (AKI) after transcatheter aortic valve implantation (TAVI) is a serious complication which is associated with increased mortality. The RenalGuard system was developed to reduce the risk of AKI after contrast media exposition by furosemide-induced diuresis with matched isotonic intravenous hydration. The aim of this study was to examine the effect of the RenalGuard system on the occurrence of AKI after TAVI in patients with chronic kidney disease.

**Methods:**

The present study is a single-center randomized trial including patients with severe aortic valve stenosis undergoing TAVI. Overall, a total of 100 patients treated by TAVI between January 2017 and August 2018 were randomly assigned to a periprocedural treatment with the RenalGuard system or standard treatment by pre- and postprocedural intravenous hydration. Primary endpoint was the occurrence of AKI after TAVI, and secondary endpoints were assessed according to valve academic research consortium 2 criteria.

**Results:**

Overall, the prevalence of AKI was 18.4% (*n = *18). The majority of these patients developed mild AKI according to stage 1. Comparing RenalGuard to standard therapy, no significant differences were observed in the occurrence of AKI (RenalGuard: 21.3%; control group: 15.7%; *p = *0.651). In addition, there were no differences between the groups with regard to 30-day and 12-month mortality and procedure-associated complication rates.

**Conclusion:**

In this randomized trial, we did not detect a reduction in AKI after TAVI by using the RenalGuard system. A substantial number of patients with chronic kidney disease developed AKI after TAVI, whereas the majority presented with mild AKI according to stage 1 (ClinicalTrials.gov number NCT04537325).

**Graphical abstract:**

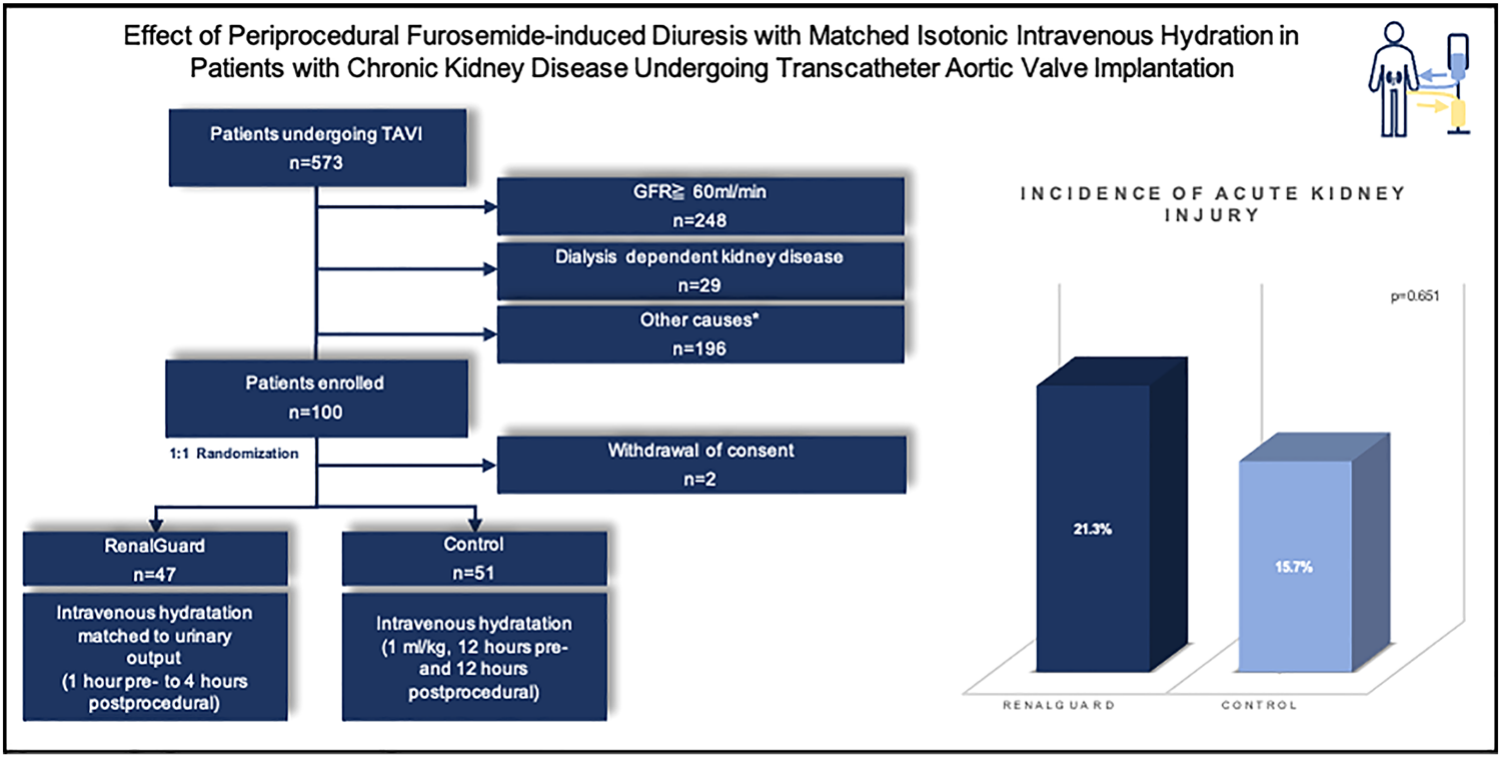

## Introduction

Acute kidney injury (AKI) is a serious complication after various cardiac interventions, as well as transcatheter aortic valve implantation (TAVI). The incidence of AKI after TAVI ranges between 8 and 57%, depending on the used definition [1]. Several studies have shown that the occurrence of AKI after TAVI is associated with significantly increased short- and long-term mortality [1, 2]. However, strategies to prevent this serious complication are scarce, and most are not widely successful. One example is the treatment with N-acetylcysteine, which initially raised high expectations [3]. Intravenous hydration before and after cardiological interventions has become the only established method in clinical routine to prevent AKI [4, 5]. However, this is particularly limited in cardiac decompensated patients, as we often see with high-grade aortic valve stenosis. For this purpose, the RenalGuard system (RenalGuard Solutions, Inc., Milford, Massachusetts) was developed, which adapts intravenous hydration to the patient's urine output. Hereby, a dilution of the contrast agent is achieved, which is intended to decrease the nephrotoxic effect by reducing the time of exposure to the tubular cells [6]. A number of studies have already shown that the occurrence of AKI after contrast-enhanced procedures is significantly reduced by the RenalGuard system, but the majority of these studies investigated AKI after coronary procedures [6–8]. Randomized trials examining the impact of RenalGuard on the occurrence of AKI after TAVI are sparse [9, 10]. Furthermore, whether the RenalGuard system reduces AKI in patients with chronic kidney disease (CKD) undergoing TAVI is not yet sufficiently known, although this patient group in particular is at high risk for AKI after TAVI [1, 11]. The aim of this randomized single-center study was to evaluate the impact of the RenalGuard system on the occurrence of AKI after TAVI in patients with CKD.

## Methods

### Study design

The present study is a single-center, open-label, randomized trial, which investigates the effect of the RenalGuard system on the occurrence of AKI after TAVI in patients with CKD. Randomization, procedure, data collection, and statistical analysis were performed at the University Heart and Vascular Center Hamburg. The study was conducted after approval by the Hamburg Ethics Committee (ethics committee approval number: PV5371), in accordance with the Declaration of Helsinki and good clinical practice. The study was registered at clinicaltrials.gov (NCT04537325), and outcomes were assessed according to the intention-to-treat design.

### Patient population

Patients admitted to the University Heart and Vascular Center Hamburg for treatment of severe aortic valve stenosis by TAVI have been screened for the study since January 2017. Inclusion criteria for the present study were (i) chronic kidney disease with an estimated glomerular filtration rate (eGFR) < 60 ml/min, (ii) severe aortic valve stenosis undergoing TAVI, and (iii) written informed consent from the patients. Excluded from the study were patients with chronic kidney disease requiring dialysis and (ii) patients with hemodynamic instability with the need for urgent treatment.

### Randomization and TAVI-procedure

Eligible patients were randomized 1:1 to periprocedural therapy with the RenalGuard system or the standard procedure at our center, a pre- and postprocedural therapy with infusion of isotonic saline solution (1 ml/kg). Potentially nephrotoxic drugs, such as metformin, were stopped 48 h prior TAVI; drugs affecting kidney function, such as angiotensin-converting enzyme inhibitors, angiotensin-receptor blockers, and mineral corticoid receptor antagonists, were discontinued on the day of the TAVI procedure. Patients in the RenalGuard group were connected to the system 1 h before TAVI. The RenalGuard system consists of a measuring unit that records the amount of fluid infused and the amount of urine excreted, the latter by using a scale on the RenalGuard device. In addition, the system displays the urine flow rate, which can be influenced by diuretic administration. The fluid was infused via intravenous access with an initial fluid bolus of 250 ml as recommended by the manufacturer and all patients received a transurethral catheter. In hybrid operating room, intravenous diuretics (furosemide) were administered to increase the urine flow rate. The target urine flow rate was above 300 ml/h, which was to be maintained throughout the procedure. After the TAVI procedure, RenalGuard therapy was continued for 4 h at the intensive care unit.

**Fig. 1:**
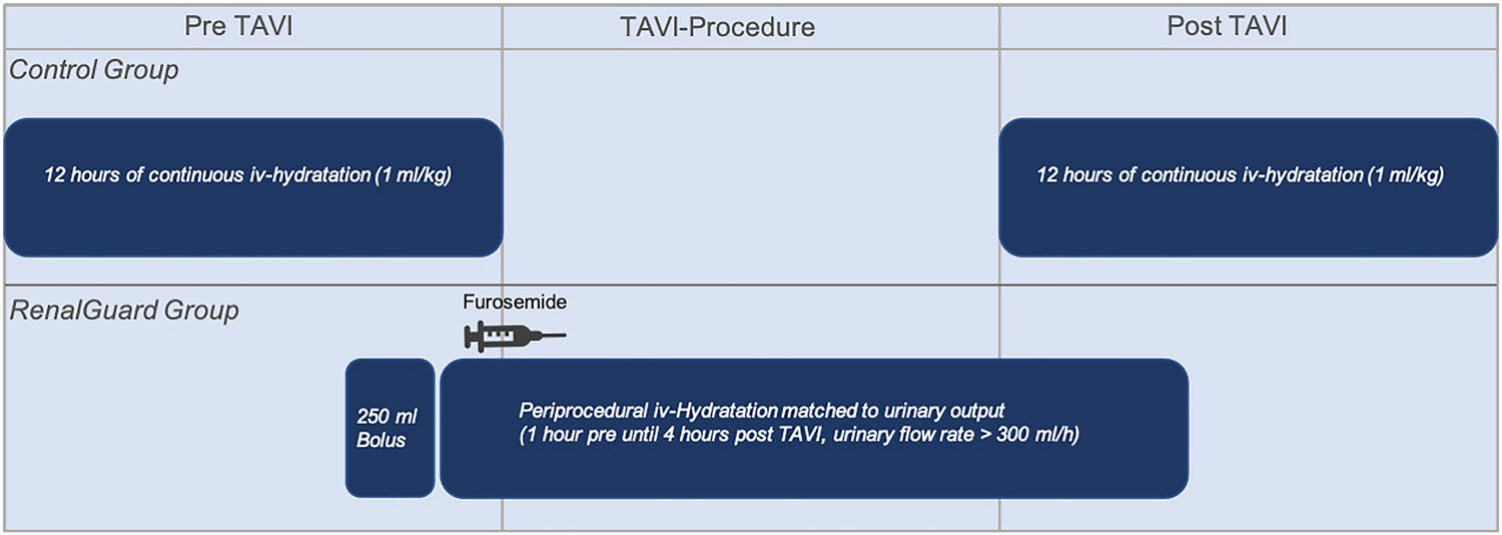
Schematic illustration of the study arms with the respective periprocedural treatment. *TAVI* Transcatheter aortic valve implantation

Patients in the control group received intravenous hydration 12 h before and 12 h after TAVI. The detailed process is shown in Fig. 1. The choice of transcatheter heart valve prosthesis and access site was determined in advance by the heart team. The TAVI procedure was performed according to best clinical practice. As part of intraprocedural monitoring of patients by the anesthesiologist, blood gas analysis was performed. If necessary, patients also received blood transfusions during TAVI. Transfusion triggers were a hemoglobin (Hb) level < 8 g/dL, symptomatic anemia, or a rapid drop in Hb in the setting of an intraprocedural bleeding event. Notably, in patients with known coronary artery disease, the indication for blood transfusion was less restrictive.

### Endpoints

The primary endpoint was the occurrence of AKI after TAVI, defined by the creatinine-based Kidney Disease: Improving Global Outcomes (KDIGO) criteria [12]. Hence, AKI is present from an increase in serum creatinine of more than 0.3 mg/dl within 48 h or of more than 150% within 7 days. Three stages of AKI were differentiated: stage 1 with an increase in serum creatinine of greater than or equal to 0.3 mg/dl within 48 h or 150–199% within 7 days; stage 2 with an increase in serum creatinine of 200–299% within 7 days, and stage 3 with a serum creatinine rise to greater than or equal to 4.0 mg/dl with an increase of at least 0.5 mg/dl within 48 h or an increase in serum creatinine of greater than or equal to 300% within 7 days or initiation of kidney replacement therapy.

Secondary endpoints were defined according to the valve academic research consortium 2 criteria and implemented 30-day and 12-month mortality and dialysis requirement after TAVI, among others [13]. In addition, recovery of kidney function was defined as a decrease of serum creatinine at discharge to within 20% of baseline serum creatinine [14].

### Biomarkers

To assess kidney function, serum biomarkers were determined before TAVI, on the day of the procedure, and postprocedural continuously until patient discharge. These included serum creatinine, urea, cystatin C, and eGFR, which was calculated according to the 2009 Chronic Kidney Disease Epidemiology Collaboration (CKD-EPI) formula for creatinine [15]. In addition, a urine biobank was created with samples from each patient before TAVI and one day after TAVI to assess kidney function with parameters including Neutrophil gelatinase-associated lipocalin (NGAL). Furthermore, electrolytes were frequently determined in both serum and urine before and after TAVI, especially sodium and potassium. Based on these values, fractional sodium excretion after TAVI was calculated.

### Statistical analysis

The sample size was estimated to achieve the primary end point of incidence of AKI after TAVI. This was based on the results of the study by Barbanti et al., and thus, we estimated an incidence of AKI in the RenalGuard group of 5% and in the control group of 25% [9]. A total of 94 patients were calculated to need to be included to achieve statistical power of 80% and to detect this difference with a two-sided significance level of 0.05. After allowing for a rate of patients with missing data or withdrawal of consent of 5%, we calculated a sample size of 100 patients, which was considered sufficient to evaluate the primary end point. Continuous variables were shown as means ± standard deviation and compared using ANOVA test. Binary variables were shown as counts (frequencies) and group differences were tested using the χ2 test.

Missing data were handled by chained-equation multiple imputation (100 imputed data sets; R package mice). Predictive mean matching was the selected method to impute missing values. All the variables shown in Tables 1, 2, and 3 were used for the multiple imputation except of variable macrohematuria due to almost constant values. Prior to imputation highly incomplete variables with 70% or higher amount of missing values as well as constant variables were excluded from analysis. In primary endpoint, no missing values were present. A *p* value of < 0.05 was considered statistically significant. Regression models were fitted for RenalGuard versus control for various endpoints. Firth correction is applied to mitigate bias caused by rare events. Logistic regressions were calculated for binary endpoints (odds ratio), and mortality was analyzed via Cox models (hazard ratio). Corresponding 95% confidence interval (CI) is given. All analyses were performed with R statistical software version 4.0.3 (R Foundation for Statistical Computing, Vienna, Austria).

**Table 1:** Baseline characteristics

	All (*n = *98)	RenalGuard (*n = *47)	Contro (*n = *51)	*p* value
Clinical data				
Age (years)	81.3 ± 5.7	81.7 ± 4.7	81.0 ± 6.5	0.520
Male sex (%)	51 (52.0)	23 (48.9)	28 (54.9)	0.698
Arterial hypertension (%)	81 (82.7)	40 (85.1)	41 (80.4)	0.727
Coronary artery disease (%)	65 (66.3)	34 (72.3)	31 (60.8)	0.320
Previous cardiac surgery (%)	12 (12.2)	5 (10.6)	7 (13.7)	0.875
Previous pacemaker-/ICD-implantation (%)	9 (9.4)	4 (8.7)	5 (10.0)	1
Diabetes (%)	36 (36.7)	18 (38.3)	18 (35.3)	0.922
Pulmonary hypertension (%)	5 (5.1)	1 (2.1)	4 (7.8)	0.409
Previous stroke (%)	10 (10.2)	7 (14.9)	3 (5.9)	0.255
Peripheral vascular disease (%)	20 (20.4)	10 (21.3)	10 (19.6)	1
Atrial fibrillation (%)	49 (50.0)	21 (44.7)	28 (54.9)	0.419
Logistic EuroSCORE (%)	13.9 ± 10.6	14.0 ± 9.9	13.68 ± 11.2	0.874
STS-PROM (%)	5.2 ± 6.8	4.3 ± 2.3	6.2 ± 9.1	0.227
NYHA stadium (%)				
NYHA I	6 (6.2)	2 (4.4)	4 (7.8)	0.771
NYHA II	14 (14.4)	8 (17.4)	6 (11.8)	0.618
NYHA III	66 (68.0)	31 (67.4)	35 (68.6)	1
NYHA IV	9 (9.3)	4 (8.7)	5 (9.8)	1
Medication at baseline^a^ (%)				
ACE inhibitor	51 (52.0)	24 (51.1)	27 (52.9)	1
ARB	29 (29.6)	14 (29.8)	15 (29.4)	1
ARNI	0 (0)	0 (0)	0 (0)	
Beta-blocker	73 (74.5)	31 (66.0)	42 (82.4)	0.103
MRA	16 (16.3)	7 (14.9)	9 (17.7)	0.924
Loop diuretic	67 (68.4)	36 (76.6)	31 (60.8)	0.143
Thiazide diuretic	13 (13.3)	7 (14.9)	6 (11.8)	0.874
Statin	57 (58.2)	29 (61.7)	28 (54.9)	0.633
Metformin	10 (10.2)	3 (6.4)	7 (13.7)	0.387
Echocardiographic data				
LVEF (%)	49.3 ± 13.1	50.9 ± 12.2	47.8 ± 13.8	
LVEF ≥ 50%	55 (58.5)	28 (63.64)	27 (54.0)	0.461
LVEF 40–49%	9 (9.6)	4 (9.1)	5 (10.0)	1
LVEF 30–39%	15 (16.0)	4 (9.1)	11 (22.0)	0.155
LVEF < 30%	15 (16.0)	8 (18.2)	7 (14.0)	0.787
EOA (cm^2^)	0.8 ± 0.3	0.8 ± 0.2	0.9 ± 0.4	0.221
Mean aortic valve gradient (mmHg)	34.2 ± 16.8	34.7 ± 17.5	33.7 ± 16.2	0.765
Severe mitral regurgitation (%)	5 (5.2)	3 (6.5)	2 (4.0)	0.924
Severe tricuspid regurgitation (%)	9 (9.5)	6 (13.0)	3 (6.1)	0.423
Preprocedural laboratory parameters				
Serum creatinine (mg/dl)	1.5 ± 0.5	1.6 ± 0.6	1.5 ± 0.4	0.351
GFR (ml/min)	36.6 ± 10.2	36.9 ± 10.7	36.4 ± 9.7	0.803
GFR 30–60 (ml/min)	72 (73.5)	35 (74.5)	37 (72.6)	1
GFR 15–29 (ml/min)	24 (24.5)	11 (23.4)	13 (25.5)	0.996
GFR < 15 (ml/min)	2 (2.0)	1 (2.1)	1 (2.0)	1
Urea (mg/dl)	34.2 ± 16.9	30.7 ± 14.2	37.4 ± 18.5	0.102
Cystatin C (mg/l)	1.9 ± 0.6	1.9 ± 0.6	1.9 ± 0.6	0.984
NGAL (ng/ml)	96.3 ± 192.1	111.4 ± 214.0	82.3 ± 169.6	0.527
C-reactive protein (mg/l)	11.4 ± 22.2	10.7 ± 19.2	12.0 ± 24.9	0.767
Serum albumin (g/l)	27.9 ± 4.7	25.7 ± 5.7	29.6 ± 2.9	0.078
NT-proBNP (ng/l)	6170.8 ± 7705.6	4913.7 ± 6833.3	7330.2 ± 8312.1	0.163
Hemoglobine (g/dl)	11.6 ± 1.9	11.3 ± 1.9	11.9 ± 2.0	0.172

*ACE* angiotensin-converting enzyme, *ARB* angiotensin receptor blocker, *ARNI* angiotensin receptor-neprilysin inhibition, *ICD* implantable cardioverter defibrillator, *LVEF* left ventricular ejection fraction, *MRA* mineralocorticoid receptor antagonist *EOA* effective orifice area, *GFR* glomerular filtration rate, *NGAL* neutrophil gelatinase-associated lipocalin, *NT-proBNP* N-terminal pro-B-type natriuretic peptide *STS-PROM* Society of Thoracic Surgeons Predicted Risk of Mortality

^a^This does not include the entire medication, only potentially nephrotoxic drugs and drugs affecting kidney function

**Table 2:** Procedural data

	All (*N = *98)	RenalGuard (*N = *47)	Control (*N = *51)	*p* value
Transfemoral access (%)	90 (91.8)	42 (89.4)	48 (94.1)	0.624
Transaxillary access (%)	4 (4.1)	3 (6.4)	1 (2.0)	0.552
Transapical access (%)	4 (4.1)	2 (4.3)	2 (3.9)	1
Local anesthesia/semiconscious sedation (%)^a^	87 (91.6)	42 (91.3)	45 (91.8)	0.433
General anesthesia (%)	8 (8.4)	4 (8.7)	4 (8.2)	1
Procedure time (min)	102.6 ± 49.9	109.1 ± 53.2	96.4 ± 46.2	1
Contrast media amount (ml)	193.3 ± 83.4	199.1 ± 89.7	187.9 ± 77.3	0.566
Periprocedural blood transfusion (%)	10 (9.9)	8 (17.5)	1 (2.9)	0.045
Transcatheter heart valve (%)				
Edwards Sapien	35 (35.7)	17 (36.2)	18 (35.3)	1
Medtronic CoreValve	13 (13.3)	6 (12.8)	7 (13.7)	1
Symetis Acurate	32 (32.7)	15 (31.9)	17 (33.3)	1
Portico	8 (8.2)	4 (8.5)	4 (7.8)	1
Allegra	10 (10.2)	5 (10.6)	5 (9.8)	1
Hemodynamic measurements				
Pre TAVI				
LV-ESP (mmHg)	156.5 ± 29.0	158.9 ± 29.3	154.4 ± 28.7	0.482
LV-EDP (mmHg)	15.1 ± 7.8	15.9 ± 7.8	14.3 ± 7.8	0.326
Peak-to-peak transaortic gradient (mmHg)	40.7 ± 23.9	41.2 ± 26.2	40.2 ± 21.8	0.859
Mean transaortic gradient (mmHg)	40.1 ± 17.5	40.5 ± 18.7	39.80 ± 16.4	0.862
Mean PAP (mmHg)	27.4 ± 13.6	28.3 ± 13.3	26.5 ± 14.0	0.597
PCWP (mmHg)	15.9 ± 9.0	17.0 ± 9.1	15.0 ± 8.9	0.383
RAP (mmHg)	8.6 ± 6.8	8.7 ± 6.5	8.5 ± 7.1	0.89
Preprocedural CO (l/min)	4.1 ± 1.3	4.2 ± 1.3	4.0 ± 1.4	0.463
Post TAVI				
LV-ESP (mmHg)	131.5 ± 23.9	130.2 ± 21.9	132.6 ± 25.7	0.645
LV-EDP (mmHg)	16.7 ± 8.3	16.8 ± 9.0	16.5 ± 7.7	0.879
Peak-to-peak transaortic gradient max (mmHg)	3.3 ± 3.1	3.4 ± 3.1	3.1 ± 3.0	0.635
Mean transaortic gradient (mmHg)	8.5 ± 4.1	8.0 ± 3.8	8.9 ± 4.3	0.241
Mean PAP (mmHg)	27.5 ± 12.1	27.6 ± 12.3	27.4 ± 11.9	0.938
PCWP (mmHg)	16.4 ± 8.6	16.1 ± 8.3	16.6 ± 8.8	0.804
RAP (mmHg)	9.8 ± 7.2	9.7 ± 6.8	9.9 ± 7.5	0.921
Postprocedural CO (l/min)	4.5 ± 1.8	4.6 ± 1.8	4.5 ± 1.8	0.897

*TAVI* transcatheter aortic valve implantation, *LV-ESP* left ventricular end-systolic pressure, *LV-EDP* left ventricular end-diastolic pressure, *PAP* pulmonary arterial pressure, *PCWP* pulmonary capillary wedge pressure, *RAP* right atrial pressure, *CO* cardiac output

^a^Combination of local anesthesia and semiconscious sedation

**Table 3:** Outcome and postprocedural data

	All (*N = *98)	RenalGuard (*N = *47)	Control (*N = *51)	*p* value
Acute kidney injury (%)	18 (18.4)	10 (21.3)	8 (15.7)	0.651
Stage 1 (%)	15 (15.3)	8 (17.0)	7 (13.7)	
Stage 2 (%)	1 (1.0)	1 (2.1)	0 (0)	
Stage 3 (%)	2 (2.0)	1 (2.1)	1 (2.0)	
Temporary dialysis (%)	1 (1.0)	1 (2.1)	0 (0)	0.967
Pacemaker implantation (%)	7 (7.1)	2 (4.3)	5 (9.8)	0.501
New onset atrial fibrillation (%)	6 (6.1)	4 (8.5)	2 (4.0)	0.633
Major bleeding (%)	11 (11.58)	8 (17.39)	3 (6.12)	0.163
Lifethreatening bleeding (%)	3 (3.16)	2 (4.35)	1 (2.04)	0.956
Disabling stroke (%)	0 (0)	0 (0)	0 (0)	
Minor vascular complications (%)	11 (11.58)	4 (8.70)	7 (14.29)	0.596
Major vascular complications (%)	7 (7.37)	6 (13.04)	1 (2.04)	0.097
Reintervention (%)	1 (1.05)	1 (2.17)	0 (0)	0.975
Postprocedural blood transfusion (%)	10 (9.7)	4 (9.5)	5 (9.9)	1
30-day mortality (%)	3 (3.1)	1 (2.2)	2 (3.9)	1
12-month mortality (%)	8 (8.4)	5 (10.9)	3 (6.1)	0.643
Total length of stay (days)	19.8 ± 40.1	15.7 ± 9.7	23.6 ± 55.0	0.349
Postprocedural length of stay (days)	13.1 ± 39.5	8.9 ± 5.5	17.1 ± 54.7	0.327
Urinary catheter-associated complications				
Urinary tract infection (%)	10 (10.2)	3 (7.4)	7 (12.8)	0.603
Macrohematuria (%)	1 (1.0)	1 (2.2)	0 (0)	0.967
Echocardiographic data				
Postprocedural LVEF (%)	51.6 ± 12.0	52.0 ± 11.1	51.1 ± 12.9	0.730
Mean aortic valve gradient after TAVI (mmHg)	8.4 ± 7.7	8.5 ± 9.2	8.3 ± 6.0	0.903
Paravalvular leakage after TAVI > mild (%)	1 (1.14)	0 (0.25)	1 (1.96)	1
Postprocedural laboratory parameters				
Maximum S-creatinine^a^ (mg/dl)	1.74 ± 0.76	1.78 ± 0.79	1.71 ± 0.74	0.614
Minimum GFR^a^ (ml/min)	34.74 ± 13.04	34.22 ± 12.76	35.21 ± 13.40	0.716
Maximum urea^a^ (mg/dl)	34.66 ± 18.68	33.25 ± 17.88	35.96 ± 19.47	0.484
Postprocedural cystatine C (mg/l)	2.0 ± 0.9	2.0 ± 0.9	2.0 ± 0.9	0.977
Postprocedural NGAL (ng/ml)	115.2 ± 195.7	118.3 ± 212.4	112.4 ± 180.0	0.901
Maximum C-reactive protein^a^ (mg/l)	85.04 ± 62.81	86.30 ± 67.59	83.88 ± 58.69	0.852
Minimum hemoglobine^a^ (g/dl)	8.8 ± 1.4	8.6 ± 1.2	8.9 ± 1.5	0.278

*LVEF* left ventricular ejection fraction, *TAVI* transcatheter aortic valve implantation, *GFR* glomerular filtration rate, *NGAL* neutrophil gelatinase-associated lipocalin

^a^All minimum and maximum values refer to the period within 7 days after TAVI

## Results

### Study population

Between January 2017 and November 2018, a total of 573 patients underwent TAVI at our center due to severe aortic valve stenosis. Of these patients, 248 had a normal kidney function with an eGFR ≥ 60 ml/min, 29 patients had chronic kidney disease requiring dialysis, and 169 patients were ineligible for the study due to other reasons, such as insufficient ability to give informed consent, language barrier, or participation in another study, resulting in a total of 100 patients enrolled in the present study. Two patients withdrew their consent, and thus, the analysis could finally be performed with 98 patients (mean age 81.3 ± 5.7; logistic EuroSCORE (logES) 13.9 ± 10.6), of which 47 patients were in the RenalGuard group and 51 patients were in the control group (central illustration). Baseline clinical and echocardiographic data are summarized in Table 1.

Overall, kidney function as investigated by eGFR and serum creatinine was markedly impaired in the entire patient population (eGFR 36.6 ± 10.2 ml/min; serum creatinine: 1.5 ± 0.5 mg/dl). Correspondingly, other biomarkers used to determine kidney function such as cystatin C and urea were elevated at 1.9 ± 0.6 mg/l and 34.2 ± 16.9 mg/dl in the overall collective. There were no differences among the two groups with respect to these parameters (Table 1). Though preprocedural NGAL levels were numerically higher in the RenalGuard group, levels for both groups were comparatively low (RenalGuard group: 111.4 ± 214.0 ng/ml; control group: 82.3 ± 169.6 ng/ml).

### Procedural data

Procedural data are shown in Table 2. The majority of patients were treated by transfemoral TAVI (*n = *90, 91.8%). Four patients (4.1%) were treated via a transaxillary and 4 patients (4.1%) via a transapical approach. A total of 5 different transcatheter heart valves (THV) were implanted, with the Edwards Sapien THV being the most commonly used at 35.7% and the Symetis ACURATE THV at 32.7%. Medtronic CoreValve THV was implanted in 13.3%, Portico THV in 8.2%, and Allegra THV in 10.2%. The total amount of contrast media was 193.3 ± 83.4 ml, and there was no significant difference between the groups in this respect (RenalGuard group: 199.1 ± 89.7; control group: 187.9 ± 77.3; *p = *0.566). In addition, there was no significant difference between the different THV systems in this regard (Edwards Sapien THV: 176.8 ± 79.5 ml, Symetis Acurate THV: 209.3 ± 90.7 ml, Medtronic CoreValve THV: 181.6 ± 80.8 ml, Portico THV: 204.0 ± 64.3 ml, Allegra THV: 206.1 ± 88.6 ml; *p = *0.64). Patients treated with the RenalGuard system received a total of 2135.3 ± 975.4 ml of fluid periprocedurally via the system and 26.5 ± 21.0 mg of furosemide. Total urine output was 1901.36 ± 972.33 ml. Notably, patients in the RenalGuard group required significantly more periprocedural red blood cell transfusions (RenalGuard group: 8 (17.5%); control group: 1 (2.9%); *p = *0.045). Results of the invasive hemodynamic measurements are also shown in Table 2. Peak-to-peak transaortic gradient was reduced from a total of 40.7 ± 23.9 mmHg before TAVI to 3.3 ± 3.1 mmHg after TAVI and cardiac output (CO) improved from 4.1 ± 1.3 l/min to 4.5 ± 1.8 l/min after TAVI. Overall, there were no significant differences in hemodynamic measurement results between the two groups; in particular, filling pressures (right atrial pressure and left ventricular end-diastolic pressure) were comparable in the RenalGuard group and the control group.

### Primary endpoint and changes in kidney function

**Fig. 2:**
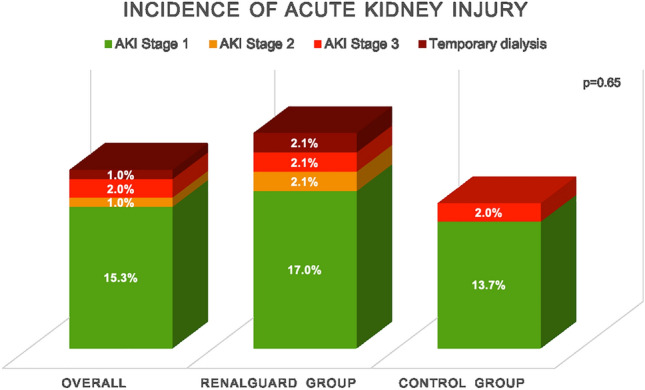
Incidence and distribution of AKI stages in the overall patient population, in the RenalGuard group, and in the control group. *AKI* Acute kidney injury

Overall, AKI occurred in 18 patients (18.4%) after TAVI. Only one of these patients, who was in the RenalGuard group, required temporary dialysis due to multiorgan failure after complicated TAVI with pericardial tamponade and intraprocedural resuscitation. There was no significant difference in AKI rates between the two groups (RenalGuard: 10 patients (21.3%), control-group: 8 patients (15.7%); *p = *0.651). The majority of patients developed stage 1 AKI (RenalGuard group: 8 patients (80% of AKI); control group: 7 patients (87.5% of AKI). One patient in the RenalGuard group developed stage 2 AKI, and one patient in each of the two groups developed stage 3 AKI. The distribution of AKI is shown in Fig. 2. Similarly, other biomarkers used to assess kidney function, such as urea, cystatin C, and NGAL, also showed postprocedural no significant differences between the RenalGuard group and the control group (Table 3).

**Fig. 3:**
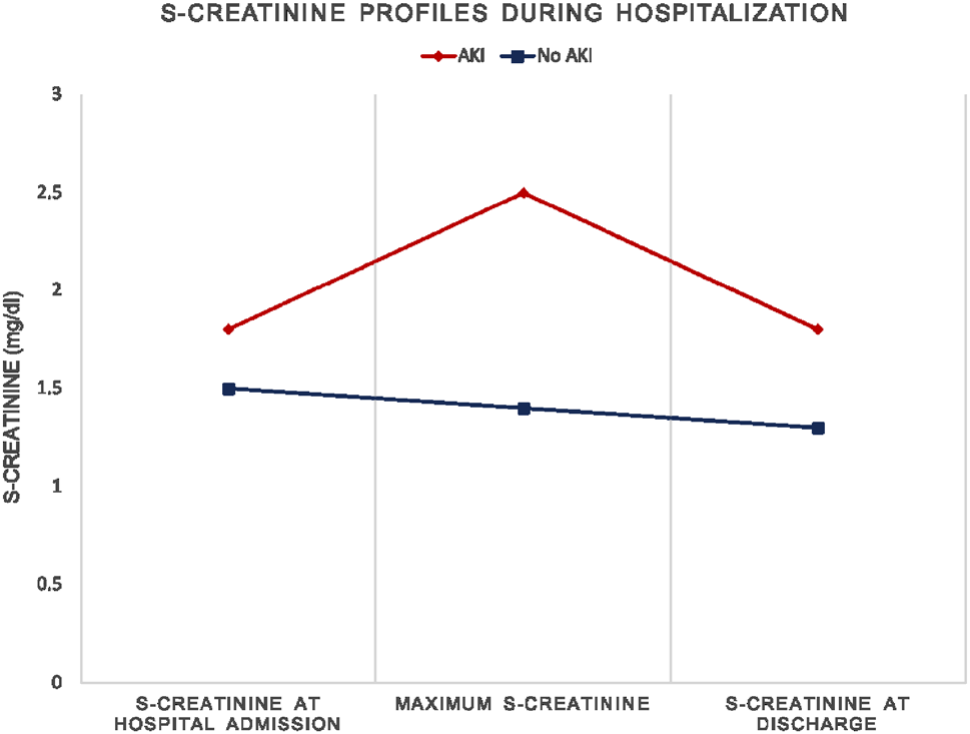
S-creatinine profiles during hospitalization in patients with and without AKI after Transcatheter aortic valve implantation. *The S-creatinine values provided are given as mean. *AKI* Acute kidney injury

Comparing patients with and without AKI after TAVI, patients with AKI had significantly higher baseline serum creatinine levels (AKI: 1.9 ± 0.8; No AKI: 1.5 ± 0.3; *p = *0.002). Biomarker analysis showed no significant differences regarding NGAL levels after TAVI between patients with and without AKI (AKI: 195.0 ± 298.2; No AKI: 97.3 ± 160.7; *p = *0.126). The additional determination of fractional sodium excretion identified that only 3 (16.7%) of the AKI patients had a value above 3%, which indicates an intrarenal origin of AKI. Analysis of creatinine profiles over hospitalization showed that most of the patients with AKI after TAVI achieved a comparable creatinine level at discharge as before TAVI. In total, 72% of patients (*n = *13) with AKI had a recovered kidney function at discharge. Notably, patients without AKI showed slightly improved creatinine levels at discharge compared to hospital admission (Fig. 3).

### Secondary endpoints

**Fig. 4:**
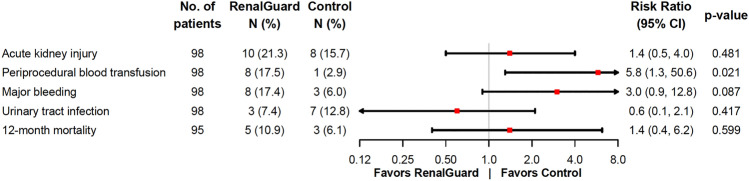
Primary and key secondary endpoints

Secondary endpoints are summarized in Table 3. There were no significant differences between the groups with regard to complication rates, for example vascular complications or pacemaker implantations. In addition, no significant differences in catheter-associated complications, such as the occurrence of urinary tract infection (RenalGuard group: 3 patients (7.4%); control group: 7 patients (12.8%); *p = *0.603) or macrohematuria (RenalGuard group: 1 patient (2.2%) and no patient in the control group; *p = *0.967), were observed. Thirty-day and 12-month mortality after TAVI were 3.1% and 8.4%, respectively, and there was no difference between the two groups (RenalGuard group: 2.2% and 10.9%; control group: 3.9% and 6.1%; *p*_1_ = 1, *p*_2_ = 0.643). The primary and key secondary end points are illustrated with their corresponding risk ratios in Fig. 4.

## Discussion

The main findings of this study are:

(i) AKI occurred in 18.4% of patients after TAVI, predominantly as a stage 1 AKI.
(ii) Kidney function in patients with AKI after TAVI recovered by discharge in the vast majority of cases.
(iii) The RenalGuard system showed no effect on the occurrence of AKI after TAVI in our patient cohort.

Patients with CKD have a particularly high risk of AKI after cardiac interventions [1, 16]. Our study confirms this, as approximately every 5th patient in our patient population was affected by AKI after TAVI. However, it must be emphasized that most of these patients had mild AKI. It is known that particularly higher AKI stages have a strong impact on mortality, whereas AKI stage 1 and recovery of kidney function are associated with a markedly better prognosis [17, 18]. Fortunately, the latter could also be observed in our patient population, as most of our patients with AKI showed a recovered kidney function at discharge. In comparison, the majority of patients without AKI had improved serum creatinine values at discharge. This finding is of importance; hence, Nijenhuis et al. showed with their study, in which they examined the 2-year outcome of 639 patients after TAVI in relation to the change in postprocedural kidney function, that patients with improved kidney function even had a better outcome than patients with stable kidney function after TAVI. Nevertheless, the worst outcome was seen in patients with AKI [19].

In the light of these data, it is apparent, why there is a strong demand for preventive approaches against AKI after cardiac procedures. The RenalGuard system provides such an approach, and initial results have been encouraging. Thus, a total of four randomized trials demonstrated a benefit of the RenalGuard system, of which three trials investigated the use of the system in elective and urgent coronary procedures and one trial after TAVI [7–9, 20]. The latter, conducted by Barbanti et al., comprised 112 patients, of whom a large proportion had a normal kidney function prior TAVI, and the incidence of AKI was 5.4% in the RenalGuard group and 25.2% in the control group (*p = *0.014). However, we cannot confirm this result with our data. Why the system was successful in this patient population and unsuccessful in a similarly large population as ours can only be speculated. A remarkable finding of our study was that patients in the RenalGuard group required significantly more frequent periprocedural red blood cell transfusions compared to patients in the control group (*p = *0.045). Since red blood cell transfusion is a well-recognized risk factor for the occurrence of AKI after TAVI, it is conceivable that the risk of AKI in the RenalGuard group increased thereby [21]. As bleeding and vascular complications were not significantly different in the two groups, it might be assumed that the increased intravenous fluid administration in the RenalGuard group resulted in dilution, which could have been to some extent misinterpreted as a bleeding situation. Of note, there was no significant difference between the two groups in terms of baseline hemoglobin levels. Another aspect that may have significantly influenced our study results regarding the effectiveness of the RenalGuard system is the etiology of AKI. The mechanism of the RenalGuard system is primarily aimed at preventing intrarenal, contrast-induced AKI [7]. However, a significant proportion of AKI after TAVI may also be of prerenal etiology or has a combined pre- and intrarenal origin [22]. This also applied to our patient collective. NGAL, which is a marker of tubular damage and intrarenal but not prerenal AKI [23], was not significantly different in patients with and without AKI after TAVI. This is confirmed by the low proportion of patients with AKI and a fractional excretion of sodium above 3% (*n = *3, 16.7% of AKI patients) as another indicator of intrarenal AKI.

Analogous to our results, the recently published STRENGTH study, which investigated the impact of the RenalGuard system on the occurrence of AKI in 259 patients after complex complex coronary, peripheral and structural interventions also failed to detect any benefit from the device [24]. Thus, the rate of AKI was 15.9% in patients treated with RenalGuard and 13.9% in patients in the control group who received pre- and post-procedural intravenous and oral hydration (*p = *0.62). As in our study, patients in the STRENGTH study had impaired preprocedural kidney function (eGFR 15–40 ml/min/m^2^). This contrasts with the study by Barbanti et al., in which patients had predominantly normal kidney function prior TAVI (eGFR 62.6 ± 25.1 mg/dl in patients treated with RenalGuard and 63.5 ± 20.6 mg/dl in patients within the control group) [9]. The contrast media amount was lower in the STRENGTH study, 116 + 68 ml (RenalGuard group) and 104 + 57 (control group), compared with the study by Barbanti et al. (RenalGuard group: 180 (140–220); control group: 170 (130–230)) and our study (RenalGuard: 199.1 ± 89.7; 187.9 ± 77.3), which is likely owed to the difference in procedures, as the STRENGTH study also included procedures with lower contrast media consumption, such as left atrial appendage closure. Considering these different study results on the RenalGuard system with both favorable and non-favorable results, the question of the consequence for clinical practice arises. Nonetheless, comparability of our study with studies that have investigated the system in coronary procedures is limited, particularly because of the differences in patient population and intraprocedural hemodynamic conditions. Given the multiple causes of AKI and the different patterns of damage, it seems reasonable to adopt a multimodal approach in the prevention of AKI after TAVI, implementing strategies against pre- and intrarenal AKI, since a "one fits all" approach seems difficult to achieve in such a heterogeneous clinical syndrome. Besides the RenalGuard system many different strategies have been investigated for prevention of intrarenal AKI after cardiac interventions. A meta-analysis by Giacoppo et al. thus examined a total of 10 different prevention strategies for contrast-induced AKI with data from a total of 124 studies. The analysis showed that statins in particular significantly reduced the risk of AKI compared with conventional saline infusion [25]. However, the current guideline mainly recommends contrast media reduction and intravenous volume administration using isotonic saline or sodium bicarbonate for the prevention of contrast-induced AKI and note that the positive results on statin therapy may be biased by the “healthy user effect” [12]. In addition, to our knowledge, no studies have yet investigated the influence of statins on the occurrence of AKI after TAVI.

While several approaches have been investigated for the prevention of intrarenal AKI, the prevention of prerenal AKI after TAVI relies mainly on the avoidance of hypotensive episodes in addition to intravenous volume administration [26]. One aspect that may also be of interest in this context is the current debate regarding the optimal timing of aortic valve replacement. Recently published data have shown that early aortic valve replacement improves patient outcome compared to conservative management [27]. As irreversible end-organ damage may occur even in the asymptomatic phase, it is imaginable that early aortic valve replacement may also reduce the risk of AKI [28]. However, whether early TAVI is beneficial, especially in patients at high risk for AKI, is yet unclear. Currently recruiting studies investigating early TAVI will probably shed light on this issue.

Additionally to the prevention of AKI, from our point of view, a precise differentiation of AKI after TAVI is useful in order to be able to react in a more targeted manner, e.g., with the help of fractional sodium excretion.

## Limitations

This study has several limitations. First, this is a single-center study with a small sample size of 100 patients; larger, multicenter data are needed to conclusively address this question. Second, the study could not be blinded, so the typical limitations of an open-label design apply to our data. Third, it cannot be excluded that the study results were influenced by contributing factors, i.e., periprocedural blood transfusions. Fourth, kidney function was monitored only until the patients were discharged; no findings can be made about the further course and any changes in kidney function after discharge.

## Conclusion

AKI after TAVI is common in a high-risk patient population with CKD; in our patient population, the incidence was 18.4%. Periprocedural therapy with the RenalGuard system did not decrease the incidence of AKI after TAVI in our patient population. Nevertheless, the majority of patients developed mild AKI and experienced full recovery of kidney function at discharge.

## Abbreviations

AKI: Acute kidney injury
CKD: Chronic kidney disease
CKD-EPI: Chronic Kidney Disease Epidemiology Collaboration
CO: Cardiac output
eGFR: Estimated glomerular filtration rate
KDIGO: Kidney Disease: Improving Global Outcomes
logES: Logistic EuroSCORE
NGAL: Neutrophil gelatinase-associated lipocalin
TAVI: Transcatheter aortic valve implantation
THV: Transcatheter heart valve
